# A recurrent de novo mutation in *ACTG1* causes isolated ocular coloboma

**DOI:** 10.1002/humu.23246

**Published:** 2017-06-06

**Authors:** Joe Rainger, Kathleen A Williamson, Dinesh C Soares, Julia Truch, Dominic Kurian, Gabriele Gillessen‐Kaesbach, Anne Seawright, James Prendergast, Mihail Halachev, Ann Wheeler, Lynn McTeir, Andrew C Gill, Veronica van Heyningen, Megan G Davey, UK10K, David R FitzPatrick

**Affiliations:** ^1^ The Roslin Institute and R(D)SVS University of Edinburgh Easter Bush Campus Midlothian UK; ^2^ MRC Human Genetics Unit, IGMM University of Edinburgh Western General Hospital Edinburgh UK; ^3^ Institut für Humangenetik Lübeck, Universitätsklinikum Schleswig‐Holstein Lübeck Germany; ^4^ Wellcome Trust Sanger Institute Genome Campus Hinxton Cambridgeshire UK

**Keywords:** ACTG1, eye development, ocular coloboma, tissue fusion

## Abstract

Ocular coloboma (OC) is a defect in optic fissure closure and is a common cause of severe congenital visual impairment. Bilateral OC is primarily genetically determined and shows marked locus heterogeneity. Whole‐exome sequencing (WES) was used to analyze 12 trios (child affected with OC and both unaffected parents). This identified de novo mutations in 10 different genes in eight probands. Three of these genes encoded proteins associated with actin cytoskeleton dynamics: *ACTG1*, *TWF1*, and *LCP1*. Proband‐only WES identified a second unrelated individual with isolated OC carrying the same *ACTG1* allele, encoding p.(Pro70Leu). Both individuals have normal neurodevelopment with no extra‐ocular signs of Baraitser–Winter syndrome. We found this mutant protein to be incapable of incorporation into F‐actin. The *LCP1* and *TWF1* variants each resulted in only minor disturbance of actin interactions, and no further plausibly causative variants were identified in these genes on resequencing 380 unrelated individuals with OC.

Ocular coloboma (OC) is a closure defect affecting epithelial tissues in the embryonic optic fissure. OC accounts for ∼11% of childhood blindness and has a birth prevalence of 0.0002–0.0005 (Morrison et al., 2002; Shah et al., 2012) and most commonly presents as the absence of iris and/or retina in the inferonasal quadrant of the eye (Nakamura, Diehl, & Mohney, 2011). OC often co‐occurs with ipsi‐ and/or contra‐lateral microphthalmia (small eye) or contralateral anophthalmia (absent eye), suggesting that these structural eye defects can represent a phenotypic continuum (Morrison et al., 2002). Many of the known OC loci encode transcription factors or signaling molecules that drive the growth of the optic cup (Chow & Lang, 2001; Zagozewski, Zhang, & Eisenstat, 2014), suggesting growth failure as a mechanism for OC occurrence, where fusion‐competent fissure margin cannot appose. Failure of epithelial fusion is another obvious mechanism, although our knowledge of the genes and pathways required for this process is limited (Brown et al., 2009). OC is proving to be genetically heterogeneous (Williamson & FitzPatrick, 2014) with no individual locus accounting for more than 3% of cases, and the majority of nonsyndromal OC‐affected individuals (>70%) have no identified genetic cause. Whole‐exome sequencing (WES) within the rare diseases component of the UK10K project (http://www.uk10k.org/) has been successful in identifying causative loci in families with OC in *YAP1* (http://www.omim.org/, MIM# 606608) (Williamson et al., 2014), *MAB21L2* (MIM# 604537) (Rainger et al., 2014), and *FZD5* (MIM# 601723) (Liu et al., 2016).

Here, we report WES on 12 trios, each comprising one affected individual with isolated OC and both unaffected parents. In three of these trios, there was a wider family history of eye malformation that would be compatible with nonpenetrance in the intervening parent. DeNovoGear analysis (Ramu et al., 2013), together with a maximum minor allele frequency in ExAC of <10^−4^, was used to identify candidate de novo mutations (DNM) among all technically robust variant calls in the affected child. Following review and Sanger sequence validation, 10 heterozygous de novo, ultrarare, plausibly disruptive variants were confirmed in 10 different genes from eight of the trios surveyed (Fig. 1, and summarized in Supp. Table S1 and Supp. Fig. S1 and have been deposited in the DECIPHER database [http://decipher.sanger.ac.uk]). Each trio was also screened for plausibly pathogenic homozygous or compound heterozygous variants in known developmental disorder genes. In proband COL5103597, a causative homozygous loss‐of‐function mutation was identified in *ADAMTS18*, which has been reported separately (Chandra et al., 2014), and no DNM were identified in this trio. No other plausible autosomal‐recessive genotypes were identified in the other trios.

**Figure 1:**
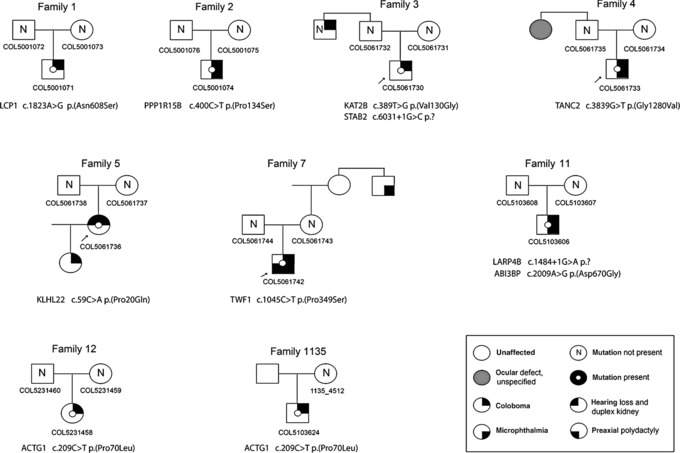
Whole‐exome de novo variant identification. Identification of 10 novel de novo variants in patients with isolated coloboma from family trios. Pedigree structures are shown with the gene variant detailed below each pedigree (chromatograms for each individual for whom DNA was available for targeted Sanger resequencing are presented in Supp. Fig. S1). Probands are indicated with an arrow.

The DNM identified in *ACTG1* (NM_001199954.1; MIM# 102560) in COL5231458 (family 12; c.209C>T (p.(Pro70Leu), RNA not analyzed)) represented a strong candidate because DNM in the ubiquitous cytoplasmic actins (encoded by *ACTB* and *ACTG1*) cause Baraitser–Winter syndrome (BWS) with OC as a prominent feature (MIM# 243310 and 614583) (Di Donato et al., 2014; Rivière et al., 2012). Review of the WES data from the remaining UK10K OC probands identified another individual, COL5103624 (family 1,135) with the identical DNM (c.209 C>T (p.(Pro70Leu), RNA not analyzed)). Clinical review confirmed that both probands had bilateral irido‐chorioretinal OC, with no involvement of the optic nerve and no evidence of the neurological and neurodevelopmental impairments that typify BWS. Neither had hearing loss. Individual COL5103624 had mild bilateral ptosis. In family 1,135, the mother did not carry the mutation, and the father was unavailable for testing.

ATP binding to actin, and subsequent hydrolysis to ADP, is coupled to conformational changes in actin monomers that appear to be essential for filament dynamics (Otterbein, Graceffa, & Dominguez, 2001) and thus actin turnover during dynamic cytoskeletal remodeling. Actin monomers have four subdomains, structurally organized so that each presents a surface interface, and arranged such that they converge around the centrally bound nucleotide. The Pro70 residue in ACTG1 is positioned N‐terminal to the previously reported BWS mutations (Fig. 2A), and is immediately adjacent to the nucleotide‐binding domain of the actin monomer, in a loop (Pro^70^‐Thr^77^) that connects the COOH‐terminal end of subdomain 2 to subdomain 1. This loop undergoes conformational reorganization upon ATP hydrolysis, where a 10^o^ rotation of subdomain 2 occurs, which in turn induces changes to interdomain interactions that affect the orientation of subdomain 4. In combination, these are predicted to have major effects on the overall structure and stability of each actin‐ADP polymer (Otterbein et al., 2001). A severe destabilizing effect of the mutant Leu70 amino acid change to the protein was predicted using FoldX (Schymkowitz et al., 2005), with a difference in free energy of folding (ΔΔG) between mutant and wild type (WT), of 6.31 kcal/mol in the ADP‐bound actin crystal structure, and ΔΔG 7.4 kcal/mol in the ATP‐bound actin crystal structure (Fig. 2B). Mouse embryonic fibroblasts (MEFs) were derived from F0 embryos following CRISPR/Cas9 gene editing to create homozygous p.Pro70Leu lines (Supp. Fig. S2). Immunofluorescence showed a marked reduction of mutant ACTG1 in filamentous F‐actin (Fig. 2C) compared with MEFs from WT littermate control embryos. We then created tetracyclin (TET)‐inducible HEK293 cell lines expressing either mutant (Leu70) or WT ACTG1 tagged with N‐terminal eGFP. Again, we observed reduced incorporation of Leu70 ACTG1 into F‐actin (Supp. Fig. S2b), whereas cosedimentation assays of these cells also showed a ∼50% reduction of Leu70 ACTG1 in the G‐actin phase compared with WT (Fig. 2D). In all cell types examined, endogenous ACTB and ACTG1 appeared to be unaffected (Fig. 2C and D; Supp. Fig. S2c). Western blotting and semiquantitative mass spectrometry on ACTG1–GFP immunoprecipitates from these HEK293 lines showed a marked reduction in the recovery of established actin‐binding partners for Leu70 ACTG1 (Fig. 2E; Supp. Table S2).

**Figure 2:**
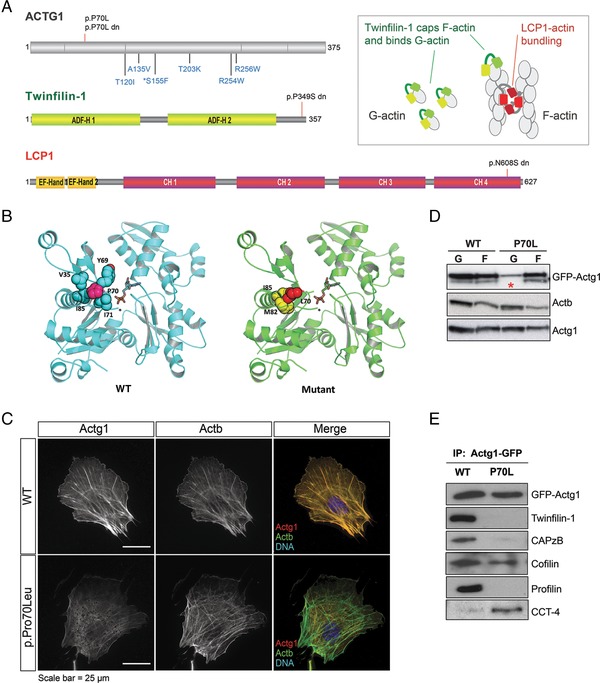
Functional effects of de novo variants on the ACTG1 interactome. **A**: A scaled cartoon illustrating the positions of the de novo variants in each protein. Domains for each protein are indicated: EF‐Hand; CH, calponin homology; ADF‐H, actin depolymerizing family homology. The LCP1 p.(Asn608Ser) variant is positioned in the fourth CH domain and the TWF1 p.(Pro349Ser) variant is in the C‐terminal tail domain of Twinfilin‐1. The p.(Pro70Leu) variant is indicated on the ACTG1 model (above), together with known Baraitser–Winter syndrome mutations (below; p.(Ser155Phe) is a recurrent variant). Inset: a schematic of the actin interactions for Twinfilin‐1 and LCP1. **B**: The intraprotein residue interactions of Pro^70^ in wild‐type (WT) ACTG1 are depicted on the crystal structure of actin bound to ADP (PDB ID: 1J6Z) on the left panel. The right panel depicts the FoldX lowest energy conformer for mutant Leu^70^ that indicates significant interatomic clashes with neighboring side chains Met^82^ and Ile^85^. In silico protein design algorithm FoldX predicted that the p.(Pro70Leu) change severely destabilizes protein structure with a ΔΔG >6.3 kcal/mol, where >1.6 kcal/mol indicates a severely destabilizing mutation. **C**: Immunofluorescence analyses on MEF cultures obtained from CRISPR/Cas9‐edited mouse embryos using antibodies specific to Actb and Actg1 indicated a reduction of F‐actin incorporation for Acgt1 Leu^70^ compared with WT. In contrast, incorporation of Actb into filaments appeared equivalent in both genotypes. **D**: Tetracyclin (TET)‐inducible HEK293 cell lines expressing either mutant Leu^70^ or WT forms ACTG1 tagged with eGFP at the N‐terminus were used for standard cosedimentation assays to separate the G‐ and F‐actin components (G and F, respectively). A significant reduction of the mutant protein was observed in the G‐actin phase (asterisk); however, levels were comparable to WT in the F‐actin phase (top). F‐ and G‐actin were unchanged for endogenous Actg1 (middle) and Actb (below). **E**: Coimmunoprecipitation assays using GFP actin as bait were subjected to mass spectrometry and showed that the p.(Pro70Leu) change affected interactions of Actg1 with multiple known actin‐interacting factors (Supp. Table S2). Western blotting confirmed these for twinfilin‐1, CAPzβ, cofilin, and profilin. In contrast, Leu^70^ enhanced interactions of Actg1 with multiple subunit components of the chaperonin containing TCP1 complex, confirmed by western blot with an anti‐CCT‐4 antibody.

Individual COL5061742 (family 7) had a DNM in *TWF1* (NM_001242397.1; MIM# 610932; c.1045 C>T; (p.(Pro349Ser), RNA not analyzed) that encodes Twinfillin‐1. However, the identification of a plausible DNM in this family is difficult to reconcile with the presence of an affected third‐degree maternal relative (Fig. 1), unless the microphthalmia in this individual is coincidental. The eye phenotype of this relative is detailed as one smaller eye and low vision, but unfortunately more detailed clinical information or DNA were not available. Twinfilins are highly conserved ubiquitous actin‐binding proteins that influence actin polymerization by forming 1:1 complexes with ADP–actin monomers to moderate F‐actin filament assembly (Palmgren, Vartiainen, & Lappalainen, 2002). Twf1 also influences the depolymerization and severing of actin filaments (Johnston, Collins, & Goode, 2015; Moseley et al., 2006). The Twinfilin‐1 p.(Pro349Ser) substitution is located at the C‐terminal “tail” region, out with the canonical actin interaction ADF‐H domains (Fig. 2A). This region was implicated in determining binding affinity to F‐actin, and complete deletion of the C‐terminal tail significantly reduces barbed‐end capping activity (Paavilainen et al., 2007). In addition, the yeast Twinfilin tail region alone can bind F‐actin, and contributes to the binding of the full‐length protein, whereas loss of the tail region significantly affects F‐actin depolymerization in vitro (Johnston et al., 2015). TET‐inducible HEK293 cells expressing the WT and mutant Twinfilin‐1 failed to identify any significant protein–protein interaction differences using immunoprecipitation with mass spectrometry (Supp. Table S2), and cosedimentation assays showed no differences in F‐/G‐actin phase distributions. However, transient transfection of FLAG‐tagged, WT ACTG1 into these HEK293 cells showed slightly increased binding of variant Twinfilin‐1 to ACTG1, compared with WT TWF1 (Supp. Fig. S2D).

Individual COL5001071 (family 1) was found to have a DNM c.1823 A>G; (p.(Asn608Ser), RNA not analyzed) in *LCP1* (NM_002298.4; MIM# 153430) that encodes LCP1 (or L‐plastin/plastin‐2), a conserved F‐actin filament cross‐linking protein of the plastin family found in structures involved in cell adhesion, motility, and invasion, such as focal adhesions, membrane ruffles, and cell projections (Janji et al., 2006). LCP1 contains two actin‐binding domains, and the p.(Asn608Ser) variant is located within the fourth of four calponin homology domains, in the second actin‐binding domain (Fig. 2A). The variant was not predicted to affect LCP1 protein stability by in silico analysis but structural modeling predicts this residue to be on the surface of the protein (Supp. Fig. S2e**)**. The variant was assessed using TET‐inducible HEK293 cells expressing WT and mutant versions of GFP‐tagged LCP1. We did not observe any reduction of protein stability (not shown), and both versions strongly associated with actin, but no clear differences in binding were observed by mass spectrometry. Cosedimentation analysis revealed these cells had increased signal in the F‐actin phase compared with WT (Supp. Fig. S2e). This may indicate abnormal LCP1–actin interactions or increased F‐actin stability in an LCP1‐Ser608 containing cellular environment.

Targeted resequencing of 380 affected individuals with OC revealed no further plausible causative variants in any of the DNM genes identified here. All alleles, with the exception of the variants in *PPP1R15B* (MIM# 613257) and *STAB2* (MIM# 608561), had ExAC allele frequencies of zero (Supp. Table S1). Using established online tools (SIFT [http://sift.bii.a-star.edu.sg/index.html] and Polyphen‐2 [http://genetics.bwh.harvard.edu/pph2/index.shtml]) to assess the biochemical impact of the encoded amino acid substitutions, only the *ABI3BP* (MIM# 606279) DNM c.2009A>G; p.(Asp670Gly) was predicted to encode a possibly damaging substitution (Supp. Table S1). ABI3BP has two fibronectin type II domains, but a role for this protein in development has not been firmly established. A recent *Abi3bp* knock‐out mouse study revealed no overt phenotype (Yang et al., 2016); however, the *ABI3BP* locus has been associated with optic disk morphology in a meta‐analysis GWAS study (Springelkamp et al., 2015). None of the other genes have been implicated in developmental eye disorders, and no ocular phenotypes have been reported in mouse knock‐out models for their orthologues.

This work strongly supports a causative role for a recurrent de novo nonsynonymous variant affecting *ACTG1* in human coloboma, although its precise role in eye development requires further investigation. It was remarkable that two other actin‐interacting proteins were implicated from the DNM screen; however, the clinical and biochemical evidence for causation is significantly less compelling, and these variants cannot currently be considered pathogenic. Trio‐based genome‐wide sequence analysis shows promise in identifying novel genetic causes and genetic mechanisms for OC, but a significantly larger number of families have to be analyzed to determine the true number of disease‐associated loci for this important eye malformation.

All datasets supporting the conclusions of this article are included within the article and the Supp. files: Table S1, Table S2, Figure S1, Figure S2, Figure S2 Legend, and Supp. Materials and Methods. The UK10K exome data are available via European Genome‐phenome Archive (https://www.ebi.ac.uk/ega/home) under the study accession number EGAS00001000127. Mass spectrometry data are available in EBI Pride repository as a ProteomeXchange submission http://www.ebi.ac.uk/pride/; (ID# PXD005090).

## Supporting information

Supporting Information

Table S1: Summary of validated de novo variants identified in UK10K Trio analyses

Table S2: Summary of quantitative heavy vs light labelled mass spectrometry data from GFP‐TRAP Co‐immunoprecipitates
