# Supplementary material for: A recurrent de novo mutation in ACTG1 causes isolated ocular coloboma
Source: Hum Mutat. 2017 Jun 6;38(8):942–6. doi: 10.1002/humu.23246 (PMC5518294; doi:10.1002/humu.23246)
Supplement: Supplementary file 1 — Supporting Information [file HUMU-38-942-s001.pdf]

Supp Mat.

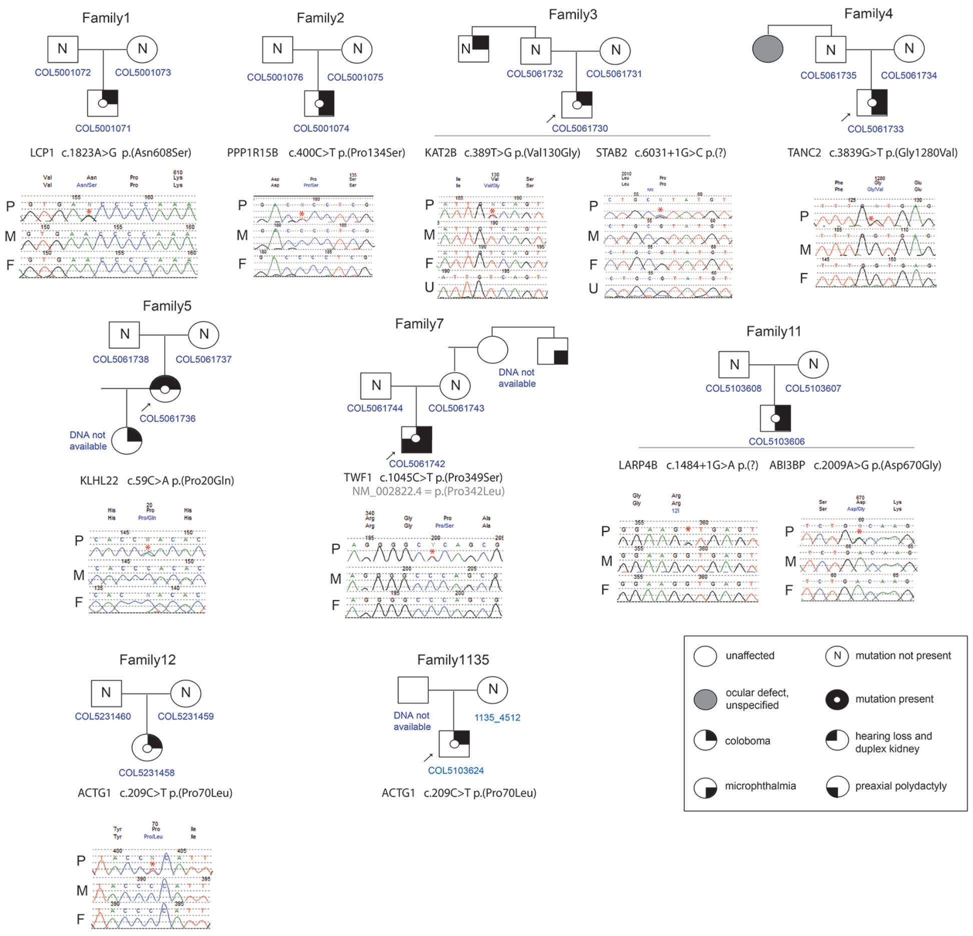

Suppl Figure S1. Identification of 10 novel *de novo* variants in patients with isolated coloboma from family trios. Pedigree structures are shown with the gene variant detailed below each pedigree and chromatograms for each individual for whom DNA was available for targeted Sanger resequencing. Probands are indicated with an arrow and variants with an asterisk. Key: P, proband; M, mother; F, father; U, paternal uncle.

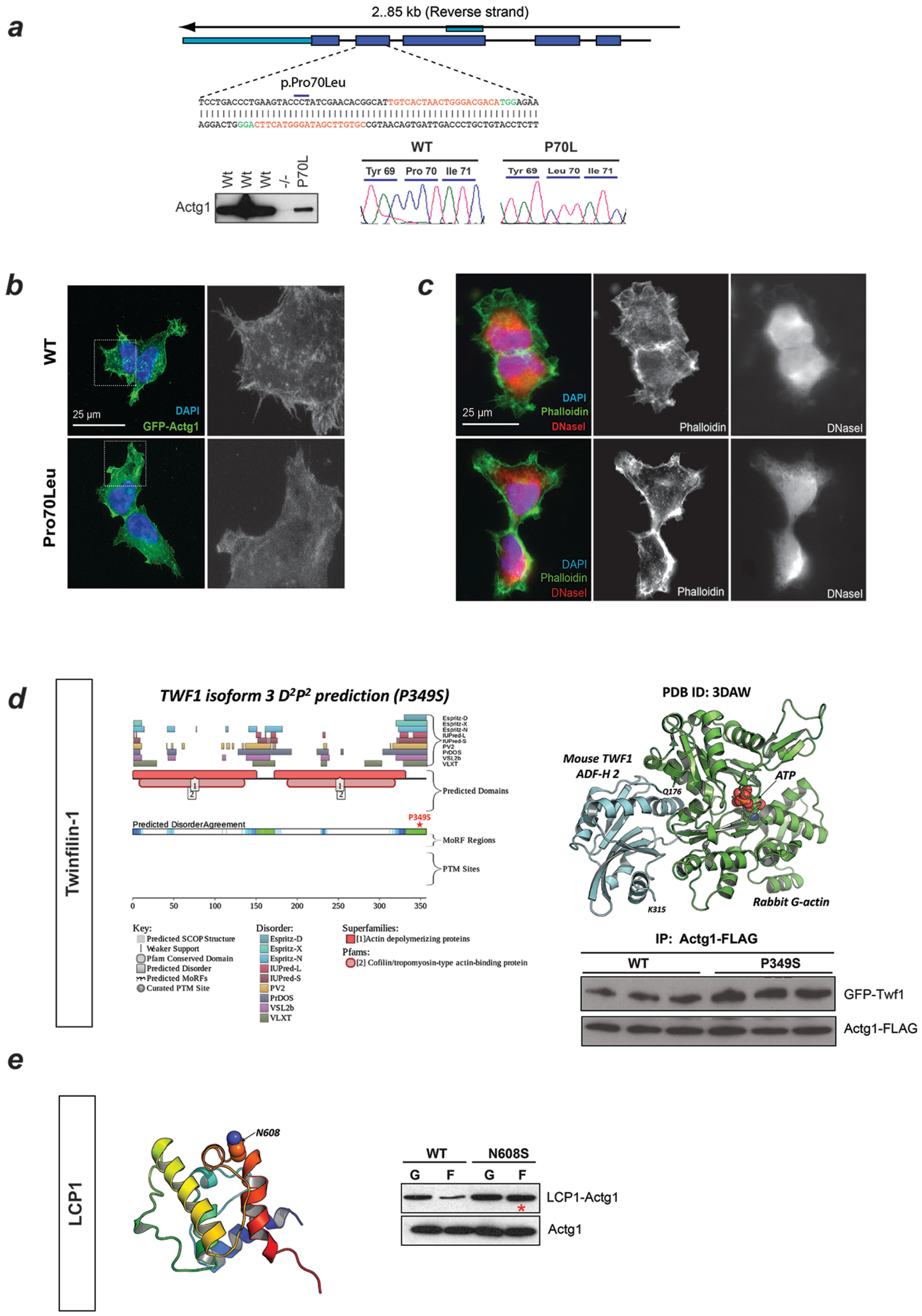

Suppl. Figure S2. (**a**) Strategy to engineer the equivalent p.(Pro70Leu) change into mouse zygotes using the CRISPR/Cas9 system. The sgRNA regions (red) and PAM sites (green) are indicated in relation to the Proline 70 codon at the *Actg1* locus. Two

guides were each cloned into SpCas9n-2A-GFP plasmids (Addgene). The repair template includes conservative nucleotide changes to reduce subsequent HDR events at successfully edited loci. These sequences are available on request. RNA was prepared and zygote injections were performed as described previously (McEntagart et al., 2015) (Ran et al., 2013b). Sanger sequencing of embryos collected at E14.5 indicated a specific homozygous p.(Pro70Leu) encoding mutation resulting from HDR of the co-injected repair template at both *actg1* alleles. Western blots using an Actg1-specific antibody against cell lysates from primary MEF cultures derived from the collected embryos. Three wild-type, one null mutation arising from a non-specific homozygous editing event, and the Actg1-Pro^{70} cell line, were used for analysis. Signal is absent from the null, but present in all other samples. (**b**) HEK293 expressing stable single-integrated CDS of either Wt or mutant Actg1 tagged with N-terminal eGFP revealed reduced Leu^{70} incorporation into F-actin, in contrast to wild type. (**c**) The mutant construct did not markedly affect endogenous F-actin or G-actin, as tested by phalloidin or DNase1 fluorophore-conjugates, respectively. (**d**) Left: *In silico* disorder predictions indicate that the C-terminal tail region of Twinfilin-1, that encompasses Proline^{349} (using the full-length Twf1 isoform NM_001242397.1) is intrinsically unstructured (consensus disorder shaded green). Right: The second ADF-homology domain of Twinfilin-1 solved in complex with G-actin is shown. The folded domain of Twinfilin-1 extends only to Lys^{315}, and therefore the location of the disordered tail region with respect to the actin-binding region is unknown. Below: Plasmid constructs expressing FLAG-Actg1 were transiently transfected into stable TET-inducible Twinfilin-1:GFP HEK293 cells and were subjected to co-immunoprecipitation against FLAG epitopes. Twf1-GFP Ser^{349} displayed increased binding to FLAG-actg1 compared to WT. (**e**) Left: the LCP1 side-chain of Asn^{608} is

exposed on the surface of the protein and therefore is unlikely to greatly destabilize the protein structure. Right: Co-sedimentation assays of TET-inducible Lcp1:GFP HEK293 cells revealed increased Ser^{608} compared to WT in the F-actin phase.

Methods

UK10K Exome Sequencing

Whole exome sequencing was performed as part of the rare disease component of the UK10K project. This study was approved by the UK Multiregional Ethics Committee (Reference: 06/MRE00/76), and informed consent was obtained from the participating families. Exome sequencing was performed as described (Ramu et al., 2013; McEntagart et al., 2015), briefly aligned using bwa 0.5.9, duplicates marked with Picard 1.43, realignment around indels and base quality scores recalibrated with GATK 1.0.5506, and variants called only with GATK Unified Genotyper. All exome data is available from European Genome-phenome Archive (https://www.ebi.ac.uk/ega) under accession EGAS00001000127. Candidate de novo mutations were identified using DeNovoGear output with a prior probability > 0.1 and were each validated using Sanger sequencing. RNA was not analysed. Nucleotide numbering uses +1 as the A of the ATG translation initiation codon in the reference sequence. The primers used for validation are available on request.

TET-Inducible Cell Lines

Multiple independent stable tetracycline-inducible Flp-In T-REx 293 (Thermo Scientific) cell lines expressing the wild-type and each variant of *Actg1*, *Twf1* and *Lcp1* as full-length N-terminal GFP-fusion proteins were derived from mouse orthologous open reading frames, as previously reported (Rainger et al., 2014).

Actin Filament Co-Sedimentation

Co-sedimentation assays were performed using the G-actin/F-actin Kit (cytoskeleton, Inc) and a TLA-100 Ultracentrifuge (Beckman). Co-immunoprecipitation was performed using the GFP-TRAP_A system (Chromotek), or using FLAG M2 beads (Sigma). The N-terminal FLAG-tagged ACTG1 construct was generated using the pJNK103 DB3.1 vector, a kind gift from Professor Alan Wright, The MRC Human Genetics Unit, Edinburgh, UK.

Mass Spectrometry

Mass spectrometry methods are presented in detail on the Pride EBI (**http://www.ebi.ac.uk/pride/**). Briefly, reductive dimethylation reactions were performed by adding formaldehyde isotopes CH_2O or CD_2O, to generate 'light' and 'heavy' labelled peptides for wild type and mutant samples respectively. Light and heavy labelled peptides were mixed for each sample set. LC-MS/MS was performed by coupling a RSLCnano LC system (Thermo Scientific) to a micrOTOF-II mass spectrometer (Bruker, Germany). Data tables are provided in Additional Table S2.

Western Blotting

For western blots, denatured and reduced protein samples were run on 4-12 % Novex Bis-Tris SDS PAGE gels with NuPAGE MOPS system reagents (Thermo Scientific). Proteins were transferred to nitrocellulose membranes and incubated with 2% BSA -TBS tween-20 (0.1 %) blocking buffer before incubation in primary antibodies overnight at 4°C. Antibodies used and their dilutions are described in the table below:

**Antibody target**	**Species**	**Manufacturer (ID number)**	**Procedure**	**Dilution**

eGFP	Mouse	Chromotek (3H9)	WB/ IF	1:2500/1/500
Cofilin	Rabbit	Santa Cruz Biotechnology (SC-33779)	WB	1:200
Twinfilin-1	Rabbit	SIGMA Prestige (HPA018116)	WB	1:200
Capzb	Rabbit	EMB Millipore (AB6017)	WB	0.1 μg/mL
Actb	Mouse	BioRad (MCA5775GA)	WB/IF	1:1000/1:500
Actg1	Mouse	BioRad (MCA5776GA)	WB/IF	1:1000/1:500
CCT4	Rabbit	Aviva Systems Biology (ARP34271)	WB	1:500
LCP1	Rabbit	Sigma-Aldrich (SAB2101327)	WB	1:200
Profilin	Rabbit	Abcam (ab180830)	WB	1:1000
Alexa Fluor 488 Phalloidin	-	Thermo Scientific (A12379)	IF	1/20
DNaseI	-	Thermo Scientific (D12372)	IF	9 μg/mL

Genome Editing and Generating Mouse Embryonic Fibroblast Cell Lines

CRISPR/Cas9 generation of p.(Pro70Leu) was performed as previously described (Ran et al., 2013a; McEntagart et al., 2015) using Guide RNAs designed to target coding exon 3 (ENSMUSE00001293379) of *Actg1* (*Actg1*-gRNA#13 5'-CGTGTTCGATAGGGTACTTC; *Actg1*-gRNA#1 5'-

TGTCACTAACTGGGACGACA). Genotyping was performed using the PCR (primers: Actg1_Fwd 5'-ACCGCGGCCTTTTCTCTC; Actg1_Rev 5'-TGTTAGCTTTGGGGTTCAGG) and Sanger sequence analysis performed using SeqMan Pro software (DNASTAR). Mouse embryonic fibroblast cell lines were generated from F0 genome edited mouse embryos at 13.5 dpc as described (Rainger et al., 2013)

Immunofluorescence

Primary MEFs were and stained for Actg1 and Actb according to Dugina et al (Dugina et al., 2009). HEKs were fixed using 3.7 % formaldehyde and rinsed three times in PBS; then incubated in primary antibodies for 2 hours and rinsed thoroughly in PBS-Tween (0.1 %). Alexa-Fluor secondary antibodies and DAPI (4',6-diamidino-2-phenylindole) were used at 1:1000 dilutions and incubated for 1 hour before being rinsed thoroughly in PBS. Coverslips were mounted in ProLong Antifade Gold (Thermo Scientific). All procedures were carried out at room temperature. Images were acquired using a Nikon Confocal A1R microscope using a 20x objective and data was acquired using NIS Elements AR software (Nikon Instruments Europe, Netherlands).

Sequence and 3-D structure analysis and in silico mutagenesis

The empirical forcefield FoldX (Guerois et al., 2002; Schymkowitz et al., 2005) was used to assess the difference in calculated free energy of folding between mutant and wildtype ACTG1 (i.e. stability change, $\Delta\Delta G$). The high resolution crystal structures of actin bound to ADP and ATP (PDB ID: 1J6Z, resolution 1.54 Å from rabbit (Otterbein et al 2000) and PDB ID: 2HF4, resolution 1.8 Å from drosophila (Rould et al., 2006) were used. The FoldX 'RepairPDB' option followed by the 'Mutate residue' was used to calculate the stability change (number of runs: 3; pH: 7;

temperature: 298 K; ionic strength: 0.05 M; VdW design: 2). The resulting mean energy change was expressed as kcal/mol. In the case of L-plastin, the NMR structure of the fourth CH domain (PDB ID: 2D85; Structural Genomics/Proteomics Initiative) was used to assess the impact of Asn608Ser, by FoldX. The crystal structure of rabbit actin bound to mouse twinfilin-1 (PDB ID: 3DAW) (Paavilainen et al., 2007)was initially assessed to locate the position of the Pro349Ser mutation. Because the C-terminal tail region is located outside the folded domain, a disorder prediction was undertaken on twinfilin-1 isoform 3 with the D^2P^2 database (Oates et al., 2013), that utilises a range of disorder predictors to highlight potential regions of disorder. Intra-protein residue interactions were determined using the Protein Interactions Calculator (PIC) (Tina et al., 2007). Inter-atomic clashes were analysed using What IF (Vriend, 1990). Surface accessibility was assessed using ASAView (Ahmad et al., 2004). PyMol (The PyMOL Molecular Graphics System, Schrödinger LLC; http://www.pymol.org) was used for 3-D visualisation, analysis and preparation of structure-based figures.

References:

Ahmad S, Gromiha M, Fawareh H, Sarai A. 2004. ASAView: database and tool for solvent accessibility representation in proteins. BMC Bioinformatics 5:51.

Dugina V, Zwaenepoel I, Gabbiani G, Clément S, Chaponnier C. 2009. Beta and gamma-cytoplasmic actins display distinct distribution and functional diversity. J Cell Sci 122:2980–8.

Guerois R, Nielsen JE, Serrano L. 2002. Predicting changes in the stability of proteins and protein complexes: A study of more than 1000 mutations. J Mol Biol 320:369–387.

McEntagart M, Williamson KA, Rainger JK, Wheeler A, Seawright A, Baere E De, Verdin H, Bergendahl LT, Quigley A, Rainger J, Dixit A, Sarkar A, et al. 2015.

Oates ME, Romero P, Ishida T, Ghalwash M, Mizianty MJ, Xue B, Dosztányi Z, Uversky VN, Obradovic Z, Kurgan L, Dunker AK, Gough J. 2013. D2P2: Database of disordered protein predictions. Nucleic Acids Res 41:.

Paavilainen VO, Hellman M, Helfer E, Bovellan M, Annila A, Carlier M-FF, Permi P, Lappalainen P. 2007. Structural basis and evolutionary origin of actin filament capping by twinfilin. Proc Natl Acad Sci U S A 104:3113–8.

Rainger J, Keighren M, Keene DR, Charbonneau NL, Rainger JK, Fisher M, Mella S, Huang JTJ, Rose L, van't Hof R, Sakai LY, Jackson IJ, et al. 2013. A Trans-Acting Protein Effect Causes Severe Eye Malformation in the Mp Mouse. PLoS Genet 9:.

Rainger J, Pehlivan D, Johansson S, Bengani H, Sanchez-Pulido L, Williamson KA, Ture M, Barker H, Rosendahl K, Spranger J, Horn D, Meynert A, et al. 2014. Monoallelic and biallelic mutations in MAB21L2 cause a spectrum of major eye malformations. Am J Hum Genet 94:915–923.

Ramu A, Noordam MJ, Schwartz RS, Wuster A, Hurles ME, Cartwright R, Conrad

DF. 2013. DeNovoGear: de novo indel and point mutation discovery and phasing. Nat Methods 10:985–987.

Ran FA, Hsu PD, Wright J, Agarwala V, Scott DA, Zhang F. 2013a. Genome engineering using the CRISPR-Cas9 system. Nat Protoc 8:2281–2308.

Ran FA, Hsu PDP, Wright J, Agarwala V, Scott D a, Zhang F. 2013b. Genome engineering using the CRISPR-Cas9 system. Nat Protoc 8:2281–2308.

Rould MA, Wan Q, Joel PB, Lowey S, Trybus KM. 2006. Crystal structures of expressed non-polymerizable monomeric actin in the ADP and ATP states. J Biol Chem 281:31909–31919.

Schymkowitz J, Borg J, Stricher F, Nys R, Rousseau F, Serrano L. 2005. The FoldX web server: An online force field. Nucleic Acids Res 33:.

Tina KG, Bhadra R, Srinivasan N. 2007. PIC: Protein Interactions Calculator. Nucleic Acids Res 35:.

Vriend G. 1990. WHAT IF: A molecular modeling and drug design program. J Mol Graph 8:52–56.
